# Development and validation of a prediction model for people with mild chronic kidney disease in Japanese individuals

**DOI:** 10.1186/s12882-024-03786-6

**Published:** 2024-10-09

**Authors:** Takahiro Miki, Toshiya Sakoda, Kojiro Yamamoto, Kento Takeyama, Yuta Hagiwara, Takahiro Imaizumi

**Affiliations:** 1 PREVENT Inc, Nagoya, Japan; 2 Center for Advanced Medicine and Clinical Research, Nagoya University Hospital, Nagoya, Aichi Japan

**Keywords:** Chronic kidney disease, Cardiovascular risk, Kidney events, Prediction model, Japanese population, Health data

## Abstract

**Background:**

Chronic kidney disease (CKD) poses significant health risks due to its asymptomatic nature in early stages and its association with increased cardiovascular and kidney events. Early detection and management are critical for improving outcomes.

**Objective:**

This study aimed to develop and validate a prediction model for hospitalization for ischemic heart disease (IHD) or cerebrovascular disease (CVD) and major kidney events in Japanese individuals with mild CKD using readily available health check and prescription data.

**Methods:**

A retrospective cohort study was conducted using data from approximately 850,000 individuals in the PREVENT Inc. database, collected between April 2013 and April 2023. Cox proportional hazard regression models were utilized to derive and validate risk scores for hospitalization for IHD/CVD and major kidney events, incorporating traditional risk factors and CKD-specific variables. Model performance was assessed using the concordance index (c-index) and 5-fold cross-validation.

**Results:**

A total of 40,351 individuals were included. Key predictors included age, sex, diabetes, hypertension, and lipid levels for hospitalization for IHD/CVD and major kidney events. Age significantly increased the risk score for both hospitalization for IHD/CVD and major kidney events. The baseline 5-year survival rates are 0.99 for hospitalization for IHD/CVD and major kidney events are 0.99. The developed risk models demonstrated predictive ability, with mean c-indexes of 0.75 for hospitalization for IHD/CVD and 0.69 for major kidney events.

**Conclusions:**

This prediction model offers a practical tool for early identification of Japanese individuals with mild CKD at risk for hospitalization for IHD/CVD and major kidney events, facilitating timely interventions to improve patient outcomes and reduce healthcare costs. The models stratified patients into risk categories, enabling identification of those at higher risk for adverse events. Further clinical validation is required.

## Introduction

Chronic kidney disease (CKD), which affects more than 10% of the world’s population, is a major noncommunicable disease that has increased mortality rates over the last two decades [1]. The high risk of cardiovascular (CV) events and premature death from cardiovascular diseases (CVD) in patients with CKD emphasizes the importance of effective disease management and prevention [1]. CKD is frequently diagnosed and treated late because it is asymptomatic in its early stages [2]. This is a global issue. The lack of early detection affects the progression of both kidney and cardiovascular complications. Therefore, screening and monitoring strategies are required. Furthermore, CKD not only lowers the quality of life but also raises medical care costs as the disease progresses, and more intensive treatment, such as dialysis and transplantation, is needed [3]. 

Over the years, several prediction models have been developed to assess cardiovascular risk, most notably the Framingham risk score (FRS) model and the Systematic Coronary Risk Evaluation (SCORE) model [4, 5]. However, these models have been reported as insufficiently accurate for predicting outcomes in patients with CKD [6, 7]. One of the primary reasons for these limitations could be that these models do not take into account CKD-specific risk factors, such as impaired kidney function and associated metabolic abnormalities. The progression of CKD is complicated and frequently accompanied by multiple comorbidities such as diabetes and hypertension, making it difficult to fit into traditional risk models [8]. The risk factors for CVD in CKD patients may include unique variables that are not typically considered in general risk assessments. For instance, factors such as estimated glomerular filtration rate (eGFR), inflammation, and vascular calcification have been determined to be significant risk factors for CVD in patients with CKD [9, 10] Consequently, several models have been developed specifically for predicting CVD onset in patients with CKD [11–13]. For example, Matsushita and colleagues used eGFR and albuminuria to predict the risk of developing CVD [9]. Chen et al. suggested that incorporating eGFR or proteinuria into the FRS model significantly improves the detection of cardiovascular events in CKD patients in stages 3 to 5 [7]. Furthermore, Shlipak and colleagues created a model for predicting cardiovascular mortality risk in patients with CKD aged 65 and up [14]. However, these models frequently rely on data that are not readily available in routine health checks, limiting their practical application.

Furthermore, most of the previously reported models focused on stages 3 and above. Current models, which are primarily designed for more advanced stages of CKD, fail to adequately address the nuances and specific needs of early-stage patients with CKD, even though patients with CKD are at a higher risk of CVD even in the early stages of CKD [15]. Even a minor decline in kidney function is associated with an increased risk of CV events and early mortality, with CV risk steadily increasing with CKD [10]. The development of this model is critical because early intervention in CKD has the potential to slow the progression of both kidney and cardiovascular disease. If early signs of CKD can be identified and addressed using risk modeling, this could lead to appropriate therapeutic intervention at an early stage, improving patients’ long-term condition and quality of life while lowering medical costs. Therefore, our model aims to predict “progression/event-prone patients” in mild CKD using readily available health check and prescription data, allowing for timely and effective intervention.

## Objective

To create and validate a new cardiovascular disease risk score for Japanese people with mild CKD.

## Method

### Study design

This is a retrospective, longitudinal cohort study of participants with mild to moderate CKD from a large Japanese database. This study used baseline and follow-up data collected between April 2013 and April 2023 from approximately 850,000 people in PREVENT Inc.’s database. The data source includes anonymized claims and health examination data provided by health insurers in Japan contracted to PREVENT Inc., as well as anonymized claims and lifestyle disease screening data from the Toyama Prefectural National Health Insurance. Originally collected for medical claims purposes, these data have been anonymized and processed to enable their use in research. The database effectively captures a diverse population of health plan members by utilizing these two distinct data sources. The dataset contains health insurance claims data, regional government administrative healthcare claims data from multiple payers, and annual employee health examination data with International Classification of Diseases, Tenth Revision (ICD-10) coding. Informed consent and ethics committee review were waived because all data in the database are anonymized and de-identified. The sample size was determined based on the need to achieve sufficient statistical power to detect significant predictors in the Cox proportional hazards model. We calculated the required sample size using the formula for time-to-event data, considering an expected event rate of 10% for outcomes over the 5-year follow-up period, a significance level of 0.05, and a power of 80%. From this calculation, the minimum number of samples was fulfilled. Given the availability of over 850,000 participants in the administrative database, we included all eligible participants to ensure robust model development and validation.

The following criteria were met for the participants of this study. ICD-10 codes were used to identify whether the patient had CKD.

- Over 18 years old.
- Individuals diagnosed with CKD with stage 2 and stage 1 disease with proteinuria (qualitative) of (+) or greater.
- Individuals in diagnosed CKD and Stage 3a.

Furthermore, individuals with a history of major adverse cardiovascular events (MACE) and/or major adverse kidney events (MAKE), as well as those who died during the follow-up period, were excluded.

This study adhered to the Transparent Reporting of a Multivariable Prediction Model for Individual Prognosis or Diagnosis (TRIPOD) reporting guidelines for prognostic studies [11]. This study’s ethics approval was waived because it used anonymized, processed data that did not identify individuals.

### Outcomes

The primary outcome was hospitalization for ischemic heart disease (IHD) or cerebrovascular disease (CVD), which included hospitalization (new or recurring) for IHD or CVD. The following ICD-10 codes were used to define these events: I20-I25 for ischemic heart disease and I60-I67, I69 for cerebrovascular disease. Secondary outcomes were major kidney events, including chronic dialysis initiation, kidney transplantation, and a new kidney failure diagnosis in this study. Specifically, the diagnosis was made using the following ICD-10 codes: N17 for acute kidney failure, N18.4 for chronic kidney disease at Stage 4, and N18.5 for chronic kidney disease at Stage 5. In addition to these diagnostic codes, specific procedure codes were also considered, including C102 for home self-peritoneal dialysis guidance and management, C155 for the addition of an automatic peritoneal dialysis device, J038 for hemodialysis (per day), J042 for peritoneal dialysis (per day), and K780-02 for living kidney transplantation.

### Predictors (risk factors)

The following risk factors were included in our analysis—age, sex, BMI, diabetes status, smoking, hypertension, dyslipidemia, systolic blood pressure (SBP), diastolic blood pressure (DBP), High-Density Lipoprotein (HDL), and Low-Density Lipoprotein (LDL) cholesterol. Candidates for these predictors were chosen based on medical expertise and availability in routine practice.

The age groups were divided into six categories as shown below. 34 and under, 35–44, 45–54, 55–64, 64–69, and 70 and up to capture the nonlinear relationship between age and the risk of cardiovascular and kidney events. These categories were chosen based on clinical practice and epidemiologic data showing different risk profiles across age groups. SBP/DBP was divided into 2 categories: SBP > 160 or DBP > 100 mmHg or not to reflect clinical thresholds commonly used to define hypertension severity and treatment targets. HDL was classified into 2 values: ≥ 40 or not. The LDL was categorized as ≥ 180 or not. These thresholds align with established guidelines for lipid management in CKD and cardiovascular risk. These variables were measured between April 2015 and March 2023. eGFR was excluded from our model for this study although previous study have reported eGFR as one of the predictive factors [16]. This is because we aimed to maintain simplicity and ensure the model’s broad applicability, particularly in settings where only routine health check data are available. Our primary goal was to create a tool that could be easily applied across various clinical settings, without the need for specialized laboratory tests like eGFR, which may not be readily accessible in all environments. By focusing on readily available data from routine health checks and administrative databases, we aimed to develop a model that can be widely used in diverse healthcare settings, ensuring its practicality and accessibility.

### Statistical analysis

#### Model derivation and development

The listwise deletion method was used to deal with missing data. In this approach, any case with missing data for any of the variables included in the analysis was excluded from the dataset. This method was chosen because the proportion of missing data was relatively low, allowing us to conduct a robust analysis without significant loss of statistical power. To test the consistency of the risk score developed, 80% of the study participants were randomly assigned to the risk prediction model as the derivation cohort, with the remaining 20% set aside to validate the risk score assigned by the derivation cohort. Data were presented as means (standard deviations) for continuous variables and counts (proportions) for categorical variables. Cox proportional hazard regression was used to calculate hazard ratios and 95% confidence intervals was used to predict hospitalization for IHD/CVD or major kidney events. Variables were selected and included that had previously been reported to be relevant.

The coefficients obtained from the Cox regression analysis were transformed into a scoring system known as a risk score [17]. A patient’s total points were calculated by adding the points assigned to their characteristics as determined by the risk score. The Cox proportional hazards regression results yielded the confidence coefficient (β) for each variable. The confidence coefficients were multiplied by 10 and assigned a score [18]. A higher number of points indicates an increased risk of hospitalization for IHD/CVD or major kidney events.

The probability of an event occurring within 5 years was also calculated using the risk score. The model was calculated using a modified version of the Framingham Heart Study [4]. In this study, the Cox regression model was initially used to estimate hazard ratios reflecting the time-to-event nature of hospitalization for IHD/CVD or major kidney events. To enhance the clinical applicability of these results, we translated the time-dependent outcomes into a 5-year risk prediction. By calculating the cumulative incidence at the 5-year mark, we provided a binary outcome (event occurring or not occurring within 5 years) that is straightforward and clinically actionable. This approach allows clinicians to easily interpret and apply the risk scores in patient care, making the model more practical for everyday use. To estimate the 5-year risk probability for hospitalization for IHD/CVD or major kidney events, the Eq. 1-s0(t)^exp(x; ib)^ was used, where S0(t) is the baseline survival function at the follow-up time (t = 5 years) and xib is a linear predictor of each participant’s total score from the fitted model. The estimated 5-year cardiovascular risk score for each category was then calculated using the average 5-year risk of hospitalization for IHD/CVD or major kidney events in that category.

#### Internal validation

In this study, internal validation was used to assess the discrimination ability of the risk models [19]. The model’s performance was measured using the concordance index (c-index), as well as the Akaike Information Criterion (AIC) and Bayesian Information Criterion (BIC) to evaluate model fit. The c-index indicates the likelihood that, given a random selection of subjects with and without the outcome, the prediction model will show a subject with the outcome [20]. To account for random bias, a 5-fold cross-validation was used [21]. The c-index, AIC, and BIC were calculated for each fold. We randomly chose those who experienced an outcome event from those who did not. The probability that each individual’s higher or lower risk score exceeds the risk score of those who experienced an event was calculated. The c-index is the ratio of correctly ordered pairs to all pairs, and a higher c-index indicates better performance. A score of 0.8 or higher was deemed excellent in this study [22]. If an outcome was missing, the patient data were removed from the analysis. Data for this study were separated for development and validation. These c-index values, along with AIC and BIC, were calculated using the 20% validation cohort, indicating the model’s predictive accuracy on unseen data. To further mitigate the risk of overfitting, we carefully selected predictors based on their relevance to hospitalization for IHD/CVD and major kidney events, informed by prior research and clinical expertise.

The statistical significance level was set at *P* < 0.05. All analyses were performed using R 4.4.0.

## Results

**Fig. 1:**
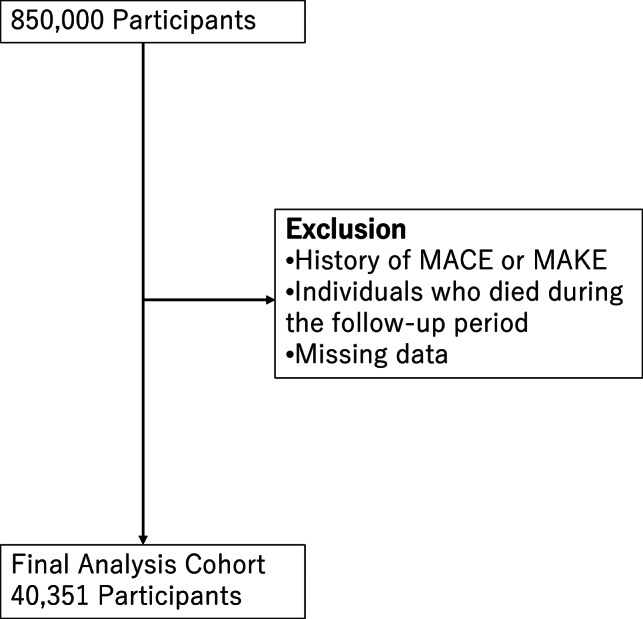
Flow chart of study participants

A total of 40,351 people were included in the study. Figure 1 shows the flowchart of this study. Table 1 contains detailed information.

**Table 1:** Baseline characteristic for the study

		CVA/IHD Event	
Characteristic	Overall, *N* = 40,351^1^	No, *N* = 38,010^1^	Yes, *N* = 2,341^1^
CKD stage			
1	2,111 / 40,339 (5.2%)	2,047 / 37,999 (5.4%)	64 / 2,340 (2.7%)
2	7,466 / 40,339 (19%)	7,079 / 37,999 (19%)	387 / 2,340 (17%)
3a	30,762 / 40,339 (76%)	28,873 / 37,999 (76%)	1,889 / 2,340 (81%)
Age (years)	59.7 (14.4)	58.9 (14.2)	71.4 (12.3)
Sex			
Men	24,747 / 40,339 (61%)	23,231 / 37,999 (61%)	1,516 / 2,340 (65%)
Woman	15,592 / 40,339 (39%)	14,768 / 37,999 (39%)	824 / 2,340 (35%)
BMI (kg/m²)	23.9 (3.8)	23.9 (3.8)	24.2 (3.7)
Hypertension (Yes)	14,245 / 40,339 (35%)	12,708 / 37,999 (33%)	1,537 / 2,340 (66%)
Dyslipidemia (Yes)	10,376 / 40,339 (26%)	9,406 / 37,999 (25%)	970 / 2,340 (41%)
Diabetes (Yes)	3,603 / 40,339 (8.9%)	3,170 / 37,999 (8.3%)	433 / 2,340 (19%)
SBP (mmHg)	125.9 (17.5)	125.5 (17.4)	131.2 (16.9)
DBP (mmHg)	76.1 (12.1)	76.2 (12.1)	75.3 (12.5)
LDL (mmHg)	124.5 (31.6)	125.0 (31.5)	116.5 (32.6)
HDL (mmHg)	60.9 (17.0)	61.2 (17.0)	56.1 (15.5)
Smoking (yes)	7,041 / 40,339 (17%)	6,674 / 37,999 (18%)	367 / 2,340 (16%)
eGFR (mL/min/1.73 m²)	60.0 (13.2)	60.2 (13.3)	57.4 (11.9)
Time of follow-up(days)	998.9 (608.2)	1,020.1 (608.9)	654.2 (478.5)

^1^Mean (SD); n / N (%)

SBP, systolic blood pressure; DBP, diastolic blood pressure, LDL, low density lipoprotein; HDL, high density lipoprotein; eGFR, estimated glomerular filtration rate; CVA, cerebrovascular accident; IHD, Ischemic heart disease

### Prediction model development for hospitalization for IHD/CVD

According to the hospitalization for IHD/CVD regression model, the risk score was assigned points based on patient characteristics (Table 2). Age significantly increased the risk score and had an impact on the risk of hospitalization for IHD/CVD in analyses based on regression models. Furthermore, male sex, smoking, diabetes, hypertension, and elevated blood pressure categories were associated with higher risk scores, emphasizing the importance of these factors in assessing cardiovascular risk in patients with mild CKD.

**Table 2:** Prediction model development for hospitalization for IHD/CVD

Variables	Coefficient	*p*-value	Score
Age 35–44 years	0.34	0.43	3
Age 45–54 years	0.87	0.037	9
Age 55–64 years	1.37	< 0.01	14
Age 65–69 years	1.98	< 0.01	20
Age 70 or more year	2.58	< 0.01	26
Men	0.57	< 0.01	6
Smoking	0.20	< 0.01	2
Diabetes	0.34	< 0.01	3
Hyperlipidemia	0.11	0.02	1
Hypertension	0.55	< 0.01	6
SBP ≥ 160mmHg or DBP ≥ 100mmHg	0.83	< 0.01	8
LDL ≥ 180	0.22	0.06	2
HDL < 40	0.23	< 0.01	2

#### Prediction model development for major kidney events

Based on the major kidney events regression model, the risk score was assigned points based on patient characteristics (Table 3). In developing the risk score model for major kidney events, our findings show that age, particularly 65 years or older, has a significant impact on the risk score, indicating an increased risk of adverse kidney outcomes. The model also identifies male sex and hypertension as significant factors associated with higher risk scores. In contrast to the model for hospitalization for IHD/CVD, smoking and certain blood pressure categories had no significant effect on the risk score for major kidney events, indicating that the risk factors for cardiovascular and kidney events are different.

**Table 3:** Prediction model development for major kidney events

Variables	Coefficient	*p*-value	Score
Age 35–44 years	0.82940783	0.42	8
Age 45–54 years	0.60460154	0.55	6
Age 55–64 years	0.61886837	0.54	6
Age 65–69 years	1.11735287	0.27	11
Age 70 or more year	1.62508859	0.10	16
Men	0.78463252	< 0.01	8
Smoking	0.04266460	0.84	0
Diabetes	0.26501446	0.21	3
Hyperlipidemia	-0.13886765	0.428	-1
Hypertension	0.77890865	< 0.01	8
SBP ≥ 160mmHg or DBP ≥ 100mmHg	-0.04233875	0.95	0
LDL ≥ 180	0.08117069	0.84	1
HDL < 40	0.34638914	0.13	3

### Incidence of event rates within 5 years in hospitalization for IHD/CVD or major kidney events

Table 4 displays the 5-year risk for hospitalization for IHD/CVD or major kidney events. The baseline survival rates at 5 years are 0.99 for hospitalization for IHD/CVD and 0.99 for major kidney events. The risk of hospitalization for IHD/CVD rises with higher scores, from a 3% probability for those with scores between 0% and 20 to 31% for those with scores of 36 or higher. Similarly, the likelihood of experiencing major kidney events within 5 years increases with the score, albeit at a slower rate, beginning at 0% for scores of 0–20 and rising to 5% for scores of 36 or higher.

**Table 4:** Estimation in 5-year risk for hospitalization for IHD/CVD or major kidney events

Score	Probability for hospitalization for IHD/CVD baseline survival rate at 5-years = 0.995	Probability for major kidney events baseline survival rate at 5-years = 0.999
Score = 〜20	3%	0%
Score = 21〜25	6%	2%
Score = 26〜30	10%	3%
Score = 31〜35	18%	5%
Score = 36 or more	31%	-

### Results of internal validation

The mean c-index for hospitalization for IHD/CVD was 0.75 (95% CI: 0.736–0.771) in the validation cohort, closely matching the mean c-index of 0.755 (95% CI: 0.751–0.759) observed in the development cohort. For major kidney events, the mean c-index in the validation cohort was 0.685 (95% CI: 0.599–0.770), compared to a mean c-index of 0.716 in the development cohort. These c-index values indicate that the model provides a robust measure of predictive accuracy, with strong performance across both cohorts.

In addition, we evaluated the model’s predictive accuracy using the c-index, Akaike Information Criterion (AIC), and Bayesian Information Criterion (BIC) across five-fold cross-validation. Tables 5 and 6 present these metrics for both hospitalization for IHD/CVD and major kidney events outcomes. For hospitalization for IHD/CVD, the mean c-index across the five folds was 0.753 (95% CI: 0.736–0.771), with AIC values ranging from 35,071.27 to 35,819.46 and BIC values from 35,149.07 to 35,891.55. These results indicate that the model has strong discriminative ability and is well-calibrated. For major kidney events, the mean c-index was 0.685 (95% CI: 0.599–0.770), with AIC values ranging from 3,094.15 to 3,396.22 and BIC values from 3,134.37 to 3,437.51. Although the predictive performance for major kidney events was somewhat lower than for hospitalization for IHD/CVD, the model still demonstrates reasonable accuracy.

**Table 5:** Model performance metrics for hospitalization for IHD/CVD

Fold	C Index (Development Cohort)	C Index (Validation Cohort)	Akaike Information Criterion (AIC)	Bayesian Information Criterion (BIC)
1	0.695829	0.748351	35469.57	35541.53
2	0.731338	0.769115	35371.52	35443.41
3	0.709754	0.749758	35616.61	35688.62
4	0.710433	0.734263	35819.46	35891.55
5	0.734127	0.765158	35077.27	35149.07
Mean	0.75538	0.753329		
Standard Error	0.001528901	0.00628557		
95%CI	0.751–0.759	0.736–0.771		

**Table 6:** Model performance metrics for major kidney events

Fold	C Index (Development Cohort)	C Index (Validation Cohort)	Akaike Information Criterion (AIC)	Bayesian Information Criterion (BIC)
1	0.695829	0.775332	3245.248	3285.937
2	0.731338	0.656506	3094.146	3134.365
3	0.709754	0.696736	3396.220	3437.510
4	0.710433	0.707068	3166.195	3206.572
5	0.734127	0.587427	3306.490	3347.558
Mean	0.716	0.685		
Standard Error	0.00721	0.00309		
95%CI	0.696–0.736	0.449–0.770		

## Discussion

This study developed and validated a new cardiovascular disease risk score for Japanese people with mild CKD by combining traditional risk factors such as age, sex, and smoking status with specific health checks and prescription data to predict the risk of hospitalization for IHD/CVD and major kidney events. The findings revealed that the developed risk score had a significant discriminatory ability for predicting hospitalization for IHD/CVD and major kidney events (mean c-index for hospitalization for IHD/CVD was 0.75 and for major kidney events was 0.69), with higher risk scores indicating an increased risk of cardiovascular and kidney events.

Previous studies have not adequately evaluated the unique risk profile of patients with mild CKD [23]. For example, the FRS and the Seattle Heart Failure Model were designed primarily for non-CKD populations and thus do not take into account risk factors unique to CKD patients [24, 25]. Therefore, these models could not predict the risk of developing cardiovascular disease in patients prone to kidney dysfunction. The model created for this study actively incorporated key risk assessment indicators in people with CKD. This enabled a more accurate prediction of cardiovascular and kidney events in people with mild CKD. The model’s internal reliability was assessed using a mean c-index of 0.75 for predicting hospitalization for IHD/CVD and 0.69 for predicting major kidney events. This score is comparable or superior to existing models [26, 27]. Furthermore, this model is considered comparable or potentially superior to existing models due to several key factors. First, this model was developed to specifically address the limitations of widely used models like the FRS and the Seattle Heart Failure Model, which were not designed for patients with CKD. By incorporating CKD-specific risk factors, our model offers a more accurate prediction of cardiovascular and kidney events in patients with mild CKD. The study by Weiner et al. aimed to assess the utility of the Framingham equations in predicting incident coronary disease specifically in individuals with CKD [6]. This study focused on a population of individuals aged 45 to 74 years without pre-existing coronary disease, using data pooled from the ARIC and CHS trials. In terms of risk prediction and discriminative ability, Weiner et al. found that the Framingham equations had poor accuracy in predicting cardiac events in CKD patients, with C-statistics of 0.62 and 0.60 for 5- and 10-year events in men, and 0.77 and 0.73 in women, respectively. This moderate discrimination suggested that the Framingham equations generally underpredict events in CKD patients. In contrast, the current study’s model achieved a mean c-index of 0.75 for predicting major adverse cardiovascular events and 0.69 for major kidney events, indicating a valid and slightly better discrimination compared to the Framingham equations, particularly for cardiovascular outcomes in CKD patients. This comparison highlights the improved predictive performance of our CKD-specific model over traditional models like the Framingham risk score.

Additionally, the model was developed using readily available data from routine health checks and administrative databases, making it practical for routine clinical use. This accessibility allows healthcare providers to seamlessly integrate the model into daily practice, even in resource-limited environments. By leveraging easily accessible data, the model enhances the management of CKD patients through early detection and targeted intervention, which can reduce healthcare costs associated with advanced treatments like dialysis and transplantation, as preventive measures are generally more cost-effective than treating advanced stages of the disease [28]. A study involving 439 CKD patients found that those classified as “high” risk by FRS were significantly more likely to experience cardiovascular events [7]. The study also showed that adding biomarkers like albumin, hemoglobin, and eGFR, along with echocardiographic parameters, improved predictive accuracy. In contrast, our study developed a new prediction model for mild CKD patients using readily available data, such as age, sex, BMI, and cholesterol levels. Unlike the FRS study, which enhanced its model with specialized markers, our model intentionally excluded such data to maintain simplicity and broader applicability. Despite this, our model achieved a strong c-index of 0.75 for predicting major adverse cardiovascular events, demonstrating robust performance that is practical for use in diverse clinical settings.

Although many countries have national policies and strategies for noncommunicable diseases, there is often a lack of specific policies focused on education and awareness regarding CKD screening, prevention, and treatment. It is crucial to enhance awareness about preventive measures among the general population, healthcare professionals, and policymakers [29]. Moreover, our model was validated using a large cohort of Japanese patients, which is a significant advantage given that many existing models were developed in non-Asian populations. This population-specific validation ensures that the model’s predictions are more accurate and relevant for Japanese individuals.

Our findings indicate that in a population with mild CKD, where traditional risk factors may not fully capture the subtle risk profile, our models can accurately predict these outcomes. In particular, by incorporating specific health check and prescription data into our models, we were able to increase their practical applicability and relevance to everyday clinical practice. This increase in model accuracy and applicability highlights the importance of a tailored approach to managing patients with CKD. Our findings highlight the potential of these models to help with early intervention strategies, particularly in identifying patients who are at higher risk of adverse outcomes and could benefit from more aggressive management or monitoring. Furthermore, the ability of our models to use routine health data provides a significant advantage. Previous studies frequently developed predictive models using variables that were not commonly used in everyday clinical practice and were difficult to obtain [30, 31]. Thus, this study attempted to use variables that are easily applicable in the real world.

The hospitalization for IHD/CVD and major kidney events risk score models were created in this study for risk assessment in patients with mild CKD. These models, which use specific health screening and prescription data, enable more accurate prediction of cardiovascular and kidney outcomes in patients with CKD. This approach not only simplifies the prediction process but also ensures that it is based on widely available data. Such an approach allows for a more personalized risk assessment for each patient, demonstrating its potential to significantly contribute to the implementation of early intervention and prevention strategies. This innovation in risk stratification has the potential to improve patient care by enabling timely and targeted management strategies for those at higher risk. Early detection enables the implementation of interventions aimed at preventing the progression of cardiovascular disease and kidney failure. Specifically, patients with high-risk scores may benefit from more intensive preventive strategies. These include making significant lifestyle changes, carefully managing blood pressure and glucose levels, and starting appropriate pharmacological treatments. For example, the dynamic nature of these models allows for continuous adjustment of patient management plans based on the changing risk profile revealed by regular health screenings and prescription data monitoring. This approach has the potential to not only improve patient outcomes but also increase the precision and responsiveness of CKD management strategies. While age plays a significant role in the predictive power of this model, the value of the model extends beyond age-related predictions for several reasons. It identifies high-risk individuals early, particularly in the elderly, allowing for timely and more effective interventions that can reduce the progression of cardiovascular and kidney disease and improve long-term outcomes. The model’s comprehensive approach is also relevant for younger patients who may be overlooked. By incorporating multiple risk factors such as sex, smoking status, diabetes, hypertension and lipid levels, the model ensures a thorough risk assessment applicable to a broad patient population.

The 5-year risk assessment of hospitalization for IHD/CVD and major kidney events in this study revealed that as scores increased, so did the incidence of both events. In particular, the risk of hospitalization for IHD/CVD increased significantly, from 3% for scores ranging from 0 to 20 to 31% for scores of 36 and higher. Conversely, major kidney events increased from 0% to a maximum of 5% over the same score range, which is significantly lower than the increased risk of hospitalization for IHD/CVD. One of the primary reasons for this difference in incidence between cardiovascular and kidney events is the outcome selection. Cardiovascular events, such as IHD or CVD, are more common and were included as endpoints, while kidney events were specifically defined as reaching kidney failure, not earlier stages like eGFR decline or doubling of creatinine, making them less frequent. This discrepancy can also be attributed to the distinct underlying physiological mechanisms. Cardiovascular events like hospitalization for IHD/CVD are often the result of atherosclerosis and the sudden blockage of blood vessels [32]. These conditions can lead to acute and severe outcomes, particularly in patients with higher risk scores. On the other hand, major kidney events tend to be the result of a gradual and progressive decline in kidney function [33], a process that generally unfolds more slowly and is less common within the timeframe of the study, particularly when the endpoint is strictly defined as kidney failure.

### Limitation

This study has several limitations. First, the study population was primarily Japanese, which may limit the results’ applicability to other ethnic groups. Differences in genetic, environmental, and lifestyle factors can change the risk profiles for CKD and cardiovascular diseases, potentially limiting the model’s global application. Second, the data we used had limitations that may have influenced how broadly our findings apply. We primarily used data from health insurance companies and government healthcare claims. These data may not accurately reflect the variety of personal and lifestyle factors that can influence CKD and its risk. Therefore, we did not account for important factors such as diet, exercise, and socioeconomic status, which could have an impact on the outcomes. Third, the use of qualitative proteinuria for identifying CKD stages 1 and 2 is another limitation. While CKD diagnostic criteria generally recommend quantitative methods, we relied on urine dipstick tests, which may be influenced by urine concentration, potentially leading to variability in diagnosis. Due to the nature of the administrative database used, no additional methods were available to confirm CKD diagnosis. Additionally, the use of ICD-10 codes in claims data introduces potential limitations related to misclassification and missing data. ICD-10 codes, being primarily used for billing, may not always capture the full clinical context, which could result in inaccuracies in identifying CKD stages or related events. Next, while the models developed in this study appear promising, they have not been tested in a real-world clinical setting. Clinical validation through prospective studies or randomized controlled trials is required to confirm the effectiveness of the model in real-world everyday clinical settings. This step is critical for ensuring that healthcare providers can rely on the model to make informed patient care decisions. Finally, the types and dosages of medications prescribed to participants throughout the follow-up period were not fully accounted for. Although certain medications are known to significantly reduce the risk of cardiovascular and kidney events, the available data did not allow these factors to be fully incorporated into the analysis. Consequently, the potential impact of these medications on the observed outcomes may not have been entirely captured. However, given that the study focused on individuals with mild CKD, the overall impact of medications may have been less significant compared to populations with more advanced stages of CKD. Nevertheless, despite these limitations, the study’s strength is its novel approach to developing a cardiovascular disease risk score specifically for Japanese individuals with mild CKD using a comprehensive dataset that includes both health insurance and government healthcare claims. It uniquely incorporates factors such as eGFR and proteinuria, which are important in the early stages of CKD, increasing the model’s predictive accuracy. Furthermore, the use of widely available routine health data makes it suitable for ongoing patient monitoring and management. Therefore, we believe this study is worth publishing. Before these clinical models can be widely implemented, additional external validation is required.

## Conclusion

In conclusion, this study created a new cardiovascular disease prediction model for patients with early CKD. We believe that our model is an important tool for managing CKD-related cardiovascular risk in patients with early CKD. Further external validation is needed to confirm the generalizability of the model.

## Data Availability

No datasets were generated or analysed during the current study.
